# Radiomics analysis of pancreas based on dual-energy computed tomography for the detection of type 2 diabetes mellitus

**DOI:** 10.3389/fmed.2024.1328687

**Published:** 2024-04-19

**Authors:** Wei Jiang, Xianpan Pan, Qunzhi Luo, Shiqi Huang, Yuhong Liang, Xixi Zhong, Xianjie Zhang, Wei Deng, Yaping Lv, Lei Chen

**Affiliations:** ^1^Department of Radiology, Liuzhou Municipal Liutie Central Hospital, Liuzhou, China; ^2^Shanghai United Imaging Intelligence Co., Ltd., Shanghai, China

**Keywords:** pancreas, dual-energy CT, type 2 diabetes mellitus, radiomics analysis, deep learning

## Abstract

**Objective:**

To utilize radiomics analysis on dual-energy CT images of the pancreas to establish a quantitative imaging biomarker for type 2 diabetes mellitus.

**Materials and methods:**

In this retrospective study, 78 participants (45 with type 2 diabetes mellitus, 33 without) underwent a dual energy CT exam. Pancreas regions were segmented automatically using a deep learning algorithm. From these regions, radiomics features were extracted. Additionally, 24 clinical features were collected for each patient. Both radiomics and clinical features were then selected using the least absolute shrinkage and selection operator (LASSO) technique and then build classifies with random forest (RF), support vector machines (SVM) and Logistic. Three models were built: one using radiomics features, one using clinical features, and a combined model.

**Results:**

Seven radiomic features were selected from the segmented pancreas regions, while eight clinical features were chosen from a pool of 24 using the LASSO method. These features were used to build a combined model, and its performance was evaluated using five-fold cross-validation. The best classifier type is Logistic and the reported area under the curve (AUC) values on the test dataset were 0.887 (0.73–1), 0.881 (0.715–1), and 0.922 (0.804–1) for the respective models.

**Conclusion:**

Radiomics analysis of the pancreas on dual-energy CT images offers potential as a quantitative imaging biomarker in the detection of type 2 diabetes mellitus.

## 1 Introduction

The anatomy of the human pancreas is closely related to its endocrine and exocrine functions. In individuals diagnosed with type 1 or type 2 diabetes, alterations in the pancreas have been observed (1). Chronic inflammation of the pancreas can cause damage to the insulin-producing cells, potentially leading to the development of diabetes. Pancreatitis and type 2 diabetes share similar risk factors (1). Several imaging techniques, such as computed tomography (CT), magnetic resonance imaging (MRI), and ultrasound (US), have been used in various studies to investigate pancreatic changes in individuals with diabetes. These studies aim to assess the size (diameter, area, or volume) as well as the fat content of the pancreas using these imaging modalities. The comparisons are made between individuals with type 1 diabetes (T1DM) and/or type 2 diabetes (T2DM) and healthy controls (2).

These imaging studies hold significant potential for providing reliable insights into diabetes mellitus (DM). Between 2008 and 2013, a total of 1,478 lean individuals without diabetes underwent CT scans (3). The presence of fatty pancreas was evaluated using a validated histological method, which measured the attenuation of the pancreas on CT scans conducted at the beginning of the study. Lower pancreas attenuation values indicate higher fat content in the pancreas (3). Furthermore, a study aimed to investigate abdominal CT biomarkers for type 2 diabetes mellitus using advanced automated deep learning techniques within a substantial clinical dataset (4). The analysis demonstrated a correlation between the diagnosis of type 2 diabetes mellitus and specific abdominal CT biomarkers, particularly measurements related to pancreatic CT attenuation and visceral fat (4). Dual-energy CT (DECT) has shown promise in providing more precise quantitative information compared to conventional methods by utilizing two different X-ray beams with distinct absorption characteristics (5).

Radiomics analysis has made significant advancements in the field of medical image analysis in recent years (6). This approach enables the extraction of numerous features from medical images, facilitating the quantification of phenotypic characteristics of tumors (7). These quantifications play a crucial role in various areas such as diagnosis, clinical prognosis, treatment selection, and decision support. By optimizing the selection of features and utilizing machine learning algorithms, it becomes possible to effectively differentiate between patients with similar outcome conditions and establish personalized prediction models based on scientific and data-driven analyses for treatment outcomes. The emerging field of radiomics also holds promise in identifying previously undetectable characteristics and assisting in the diagnosis and prediction of diabetes through pancreas imaging. For instance, a study focused on evaluating the diagnostic accuracy of a Dual-Energy Computed Tomography (DECT)-based technique that utilizes iodine quantification and fat fraction analysis for early detection of acute pancreatitis (8). The results indicated that DECT with iodine quantification exhibits higher sensitivity in diagnosing early acute pancreatitis compared to standard image evaluation methods (8). Furthermore, another study aimed to assess whether an AI model based on pancreas radiomics can identify the CT imaging pattern associated with type 2 diabetes (9). The findings showed that the model successfully detects the imaging pattern linked to type 2 diabetes. However, further enhancements and validation are necessary to evaluate its potential for identifying type 2 diabetes in the millions of CT scans conducted annually.

Our previous study investigated the clinical value of pancreatic fat fraction measured on DECT images for the detection of type 2 diabetes mellitus (10). Given the burgeoning evidence linking pancreatic imaging characteristics with DM and the advancements in imaging and analytical technologies, we hypothesize that radiomics analysis of dual-energy CT images of the pancreas can identify specific imaging biomarkers that quantitatively differentiate individuals with type 2 diabetes mellitus from non-diabetic controls. This approach aims to establish a novel, quantitative imaging biomarker for T2DM, leveraging the precision of DECT and the analytical power of radiomics to enhance early detection and intervention strategies for diabetes mellitus.

## 2 Materials and methods

### 2.1 Study population

This retrospective study received approval from the Ethics Committee of Liuzhou Municipal Liutie Central Hospital (Approval No. 2021033), and the requirement for informed consent was waived. We conducted a search in our medical information database for cases that occurred between September 2021 and July 2022. Eligible patients meeting the following criteria were included in the study: (1) those who underwent dual-energy abdominal CT with a non-contrast phase; (2) individuals who had blood tests done within 3 days before and after DECT; and (3) patients whose electronic medical records clearly indicated either “type 2 diabetes” or “non-diabetes.” Patients with malignant tumors (*n* = 12), pancreatitis (*n* = 5), or significant pancreatic atrophy without visible pancreatic parenchyma (*n* = 4) were excluded from the study.

### 2.2 Datasets

#### 2.2.1 DECT images

**Figure 1:**
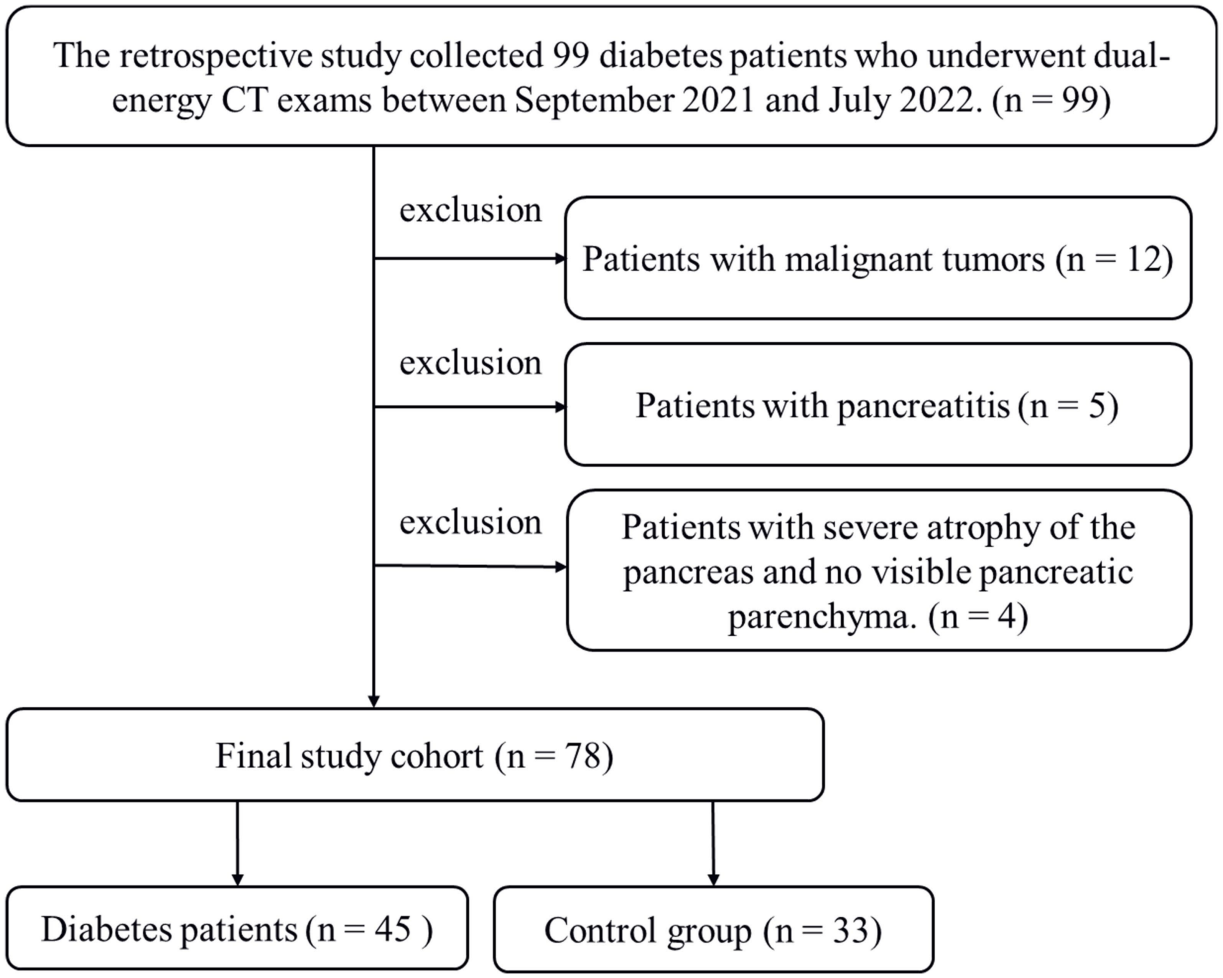
Flowchart of the study including diabetes patients enrolled with dual-energy CT images.

Abdominal DECT was conducted using a Dual-source CT scanner (SOMATOM Drive, Siemens Healthineers, Forchheim, Germany). The tube voltages used were 100 kV and Sn140 kV, with average effective tube currents of 250 mAs and 193 mAs. Automatic tube current modulation technology (CARE Dose 4D) was employed for dose control. The CT parameters were as follows: detector collimation of 32 × 0.6 mm, pitch of 0.6, gantry rotation time of 0.5 s, and matrix size of 512 × 512. Reconstruction of the CT image was performed using an iterative reconstruction technique called Adaptive Model-based Iterative Reconstruction (ADMIRE) with the Q30f algorithm, resulting in a reconstructed image thickness of 1.5 mm (see Figure 1).

#### 2.2.2 Clinical variables

The following 24 clinical data were collected: (1) categorical variables: gender, lipid turbidity index, hemolysis index, jaundice index, and clinical manifestations (now the criteria for the diagnosis of diabetes include the patient exhibiting symptoms of polydipsia, polyuria, polyphagia, and weight loss (referred to as “three excesses and one deficiency”), along with elevated blood glucose levels and the presence of glucose in the urine (which should not be found in normal urine). So if the subject has these manifestations the category of the subject is “positive”); (2) normally distributed variables: age, albumin, globulin, systolic blood pressure, total cholesterol, and high-density lipoprotein; (3) non-normally distributed variables: hematocrit, low-density lipoprotein. Others are listed in Table 1.

**Table 1:** Clinical variables of the study population.

Indicators	Control group (*n* = 33)	Diabetic group (*n* = 45)	*p*-value	Note
*Categorical variables*				
Gender, Female:Male	12:21	19:26	0.601	
Clinical manifestations None:Present	33:0	36:9	0.006	
Lipid turbidity index	6:26:1:0	4:38:2:1	0.535	0:1:2:3
Hemolysis index	20:12:0:1	36:7:2:0	0.065	0:1:2:3
Jaundice index	2:28:2:1	2:37:6:0	0.483	0:1:2:3:4
*Normal distribution variables*				
Age (years)	59 ± 14	64 ± 10	0.083	
Albumin	28.2 ± 5.0	28.6 ± 7.4	0.771	
*Globulin	40.6 ± 3.6	35.8 ± 5.0	0.000	
Systolic blood pressure	135.8 ± 22.6	137.3 ± 21.5	0.755	mmHg
Total cholesterol	4.8 ± 0.9	5.0 ± 1.6	0.416	mmol/L
High-density lipoprotein	1.3 ± 0.3	1.3 ± 0.3	0.721	mmol/L
*Non-normally distributed variables*				
Endogenous creatinine clearance rate (%)	72.2 (38.9)	90.2 (38.2)	0.004	
Hematocrit (%)	42.6 (5.2)	40.6 (9.6)	0.055	
Albumin/Globulin ratio (%)	1.4 (0.5)	1.3 (0.4)	0.005	
Low-density lipoprotein	2.7 (1.1)	2.7 (1.7)	0.903	mmol/L
Indirect bilirubin	6.7 (4.6)	7.1 (4.5)	0.980	
AST	18.0 (8.0)	17.0 (13.5)	0.598	IU/L
ALT	18.0 (9.5)	19.0 (17.5)	0.567	IU/L
*Total protein	69.5 (9.9)	63.8 (7.4)	0.002	
Triglycerides	1.3 (0.9)	1.5 (1.2)	0.482	mmol/L
Total bilirubin	12.2 (7.7)	11.9 (5.2)	0.561	μmol/L
Direct bilirubin	4.4 (2.1)	4.3 (2.7)	0.675	μmol/L
Creatinine	73.0 (17.5)	71.0 (36.0)	0.992	μmol/L
Diastolic blood pressure	78.0 (16.5)	80.0 (15.0)	0.689	mmHg

ALT, alanine transaminase; AST, aspartate transaminase; clinical manifestations, polyphagia, polydipsia, polyuria, and weight loss; categorical variables, described using sample proportions; normal distribution variables, described using mean ± standard deviation; non-normally distributed variables, described using median (interquartile range).

### 2.3 Pancreas segmentation on DECT images

**Figure 2:**
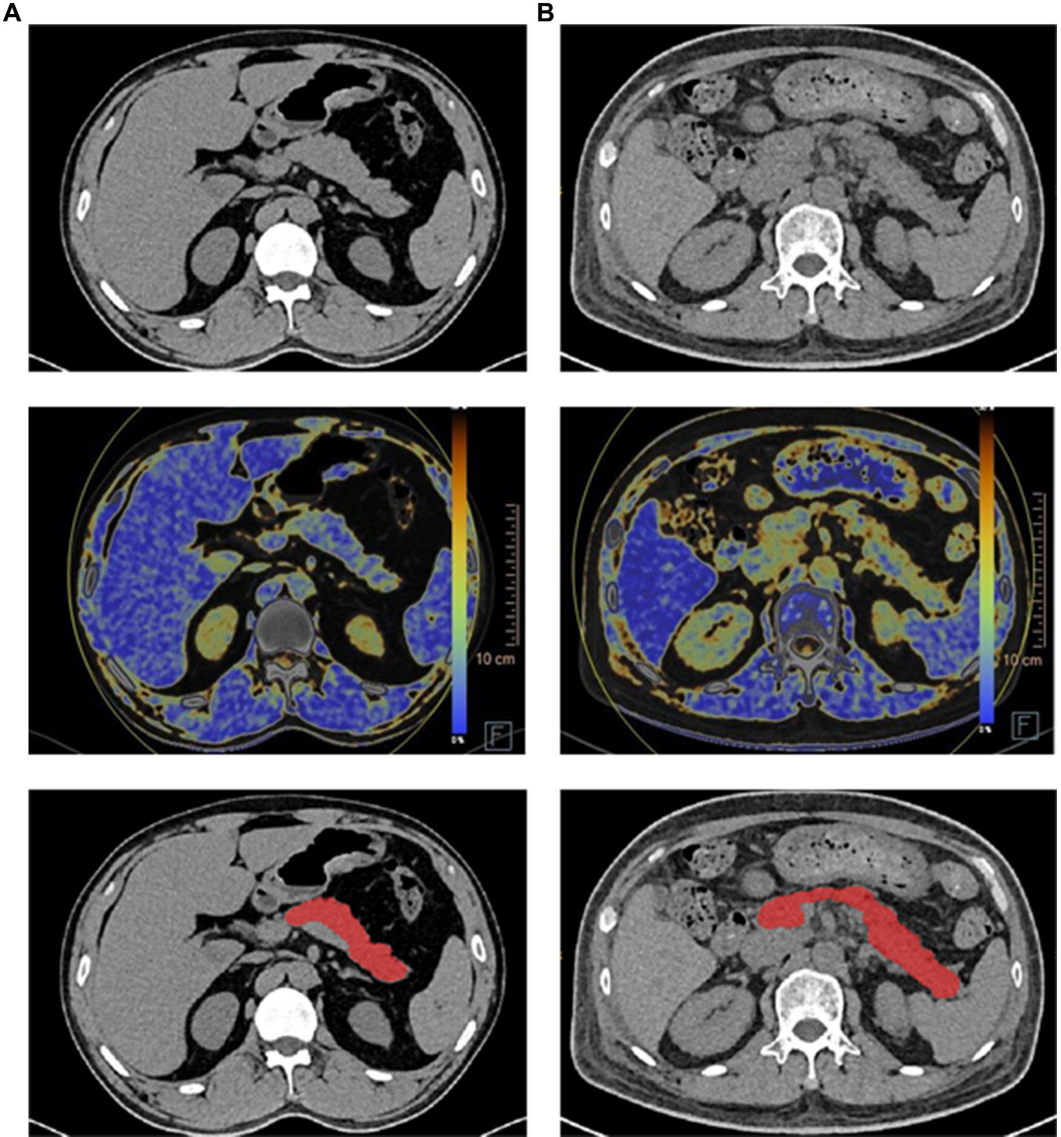
Illustration of pancreas DECT images, dual-energy fat map, and the segmentation results (each row from top to bottom) of the healthy subject and diabetes patient. **(A)** Healthy subject. **(B)** Diabetes patient.

In our previous work (11), we implemented a deep learning framework that utilized a cascade coarse-to-fine segmentation approach with an attention mechanism to segment organs. In this study, we applied this deep learning framework specifically to segment the pancreas from fusion DECT images. The segmentation process was carried out using the uAI Research Portal (United Imaging Intelligence, China) (12), which is a clinical research platform based on the Python programming language (version 3.7.3). Figure 2 displays the original pancreas DECT image and the corresponding segmentation results. In our previous work (11), we conducted a quantitative analysis comparing the DECT results between the diabetes and control groups. Figure 2 demonstrates that the head of the pancreas exhibited lower density and higher fat fraction compared to the body and tail of the pancreas in the diabetes group. Finally, all delineations were reviewed by a highly experienced chief radiologist with 8 years of expertise in abdominal imaging.

### 2.4 Radiomics extraction and selection

A total of 2,264 radiomic features were extracted from each pancreas region. These features consisted of 104 original features, which were further categorized into 18 first-order statistics, 14 shape features, and 21 texture features. The texture features included Gray-Level Co-occurrence Matrix (GLCM), Gray-Level Run-Length Matrix (GLRLM), Gray-Level Size-Zone Matrix (GLSZM), Gray-Level Dependence Matrix (GLDM), and Neighboring Gray-Tone Difference Matrix (NGTDM). Additionally, 14 image filters including Box Mean, Additive Gaussian Noise, Binomial Blur Image, Curvature Flow, Box Sigma Image, LoG with sigma values of 0.5, 1, 1.5, and 2 were applied to generate derived images. Derived images were further processed using Wavelet filters (LLL, LLH, LHL, LHH, HLL, HLH, HHL, HHH), Normalize, Laplacian Sharpening, Discrete Gaussian Mean Speckle Noise Recursive Gaussian and Shot Noise to extract first-order statistics and texture features within the pancreas regions. This resulted in a total of 2,160 derived features.

**Figure 3:**
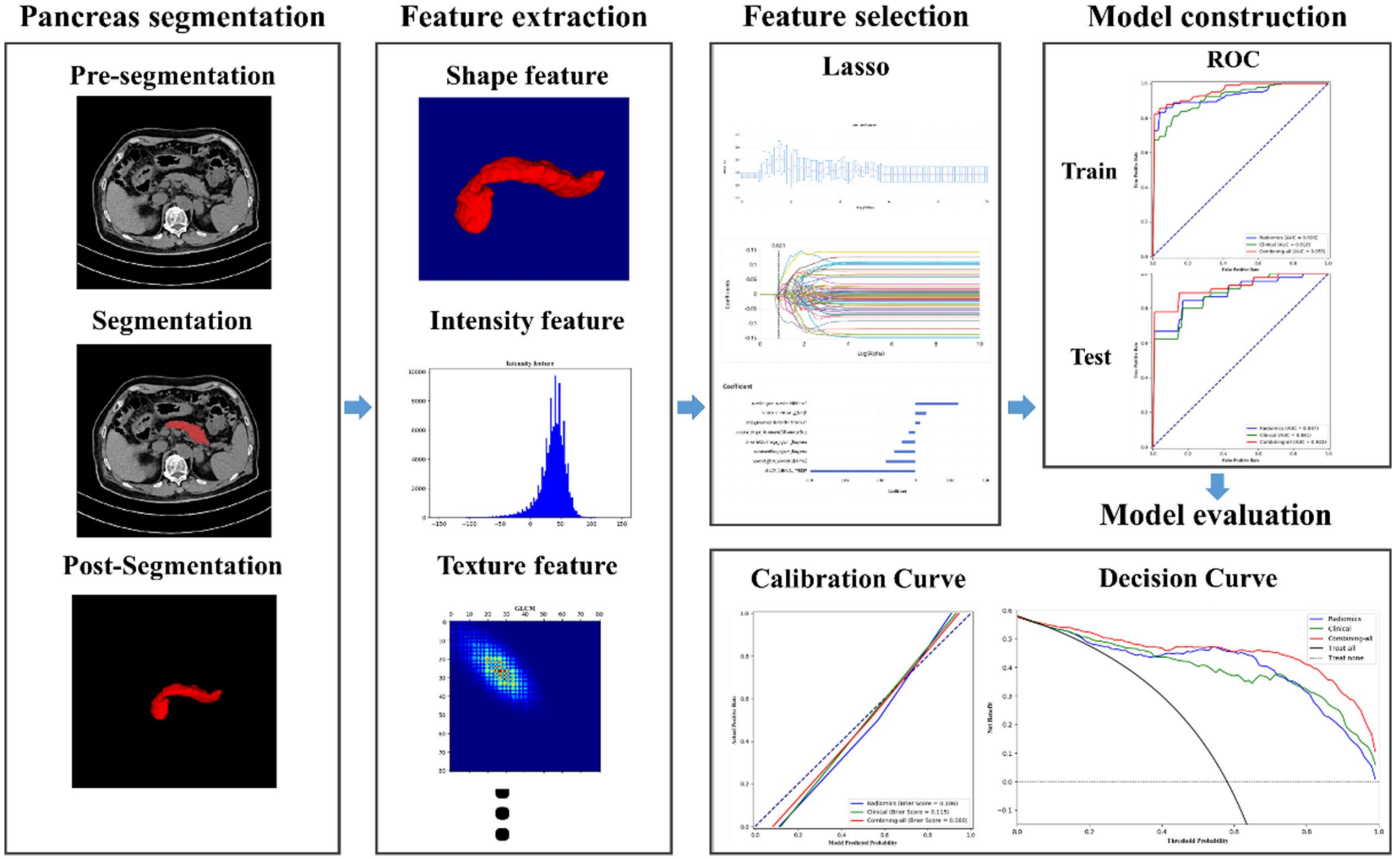
Flowchart of radiomics analysis on pancreas DECT images.

Figure 3 illustrates the flowchart of the radiomics analysis. First, radiomic features were calculated from the region of interest (ROI) on each DECT image. All the radiomic features were then normalized using *Z*-score. Next, the least absolute shrinkage and selection operator (LASSO) technique was applied to sift through these normalized radiomic features, with the aim of identifying those with the highest predictive reliability for type 2 diabetes mellitus (T2DM). This critical step focused on isolating features that are predictive of the binary outcome of diabetes presence (yes or no). Then a similar LASSO selection process was conducted for the clinical features gathered from the study participants. The radiomic and clinical features deemed significant through these selection processes were then amalgamated. A final round of LASSO selection was performed on this combined set to refine and identify a comprehensive feature set.

LASSO selection was employed to identify the most reliable predictive radiomic features. Finally, another round of LASSO selection was performed to evaluate clinical features. The selected radiomic and clinical features were merged and subjected to another round of LASSO selection to obtain a comprehensive combined feature set.

### 2.5 Model construction

Three machine learning models were developed for binary classification (diabetes or not) using three classifiers: random forest (RF), support vector machine (SVM), and logistic regression (LR). The models were built based on the selected features and/or clinical features. The input data for the detection model came from one of the three feature sets: (1) radiomic features with 7 variables, (2) clinical features with 8 variables, or (3) a combination of all features with 8 variables. To enhance performance, a grid search was performed to fine-tune the parameters for different features and classification algorithms.

### 2.6 Statistical analysis

The receiver operating characteristic curve (ROC) was generated to evaluate the performance of the detection model, and several performance metrics such as sensitivity (SEN), specificity (SPE), accuracy (ACC), F1-Score, and area under the curve (AUC) were computed. The confidence intervals for cross-validated AUC were computed to estimate the performance of each model. The demographic data was processed using the uAI Research Portal to examine significant differences in variables between the training set and the validation set. Python (version 3.6) was utilized for programming the training, validation of the prediction model, and conducting statistical analysis.

## 3 Results

Based on Harrell’s guideline, the number of selected features should be less than 10% of the sample size. Consequently, in our experiment involving the radiomic and clinical features, as well as the final combination of radiomic and clinical features, the number of selected features was less than 10% of the sample size (13, 14).

### 3.1 Assessment of radiomic and clinical features

**Figure 4:**
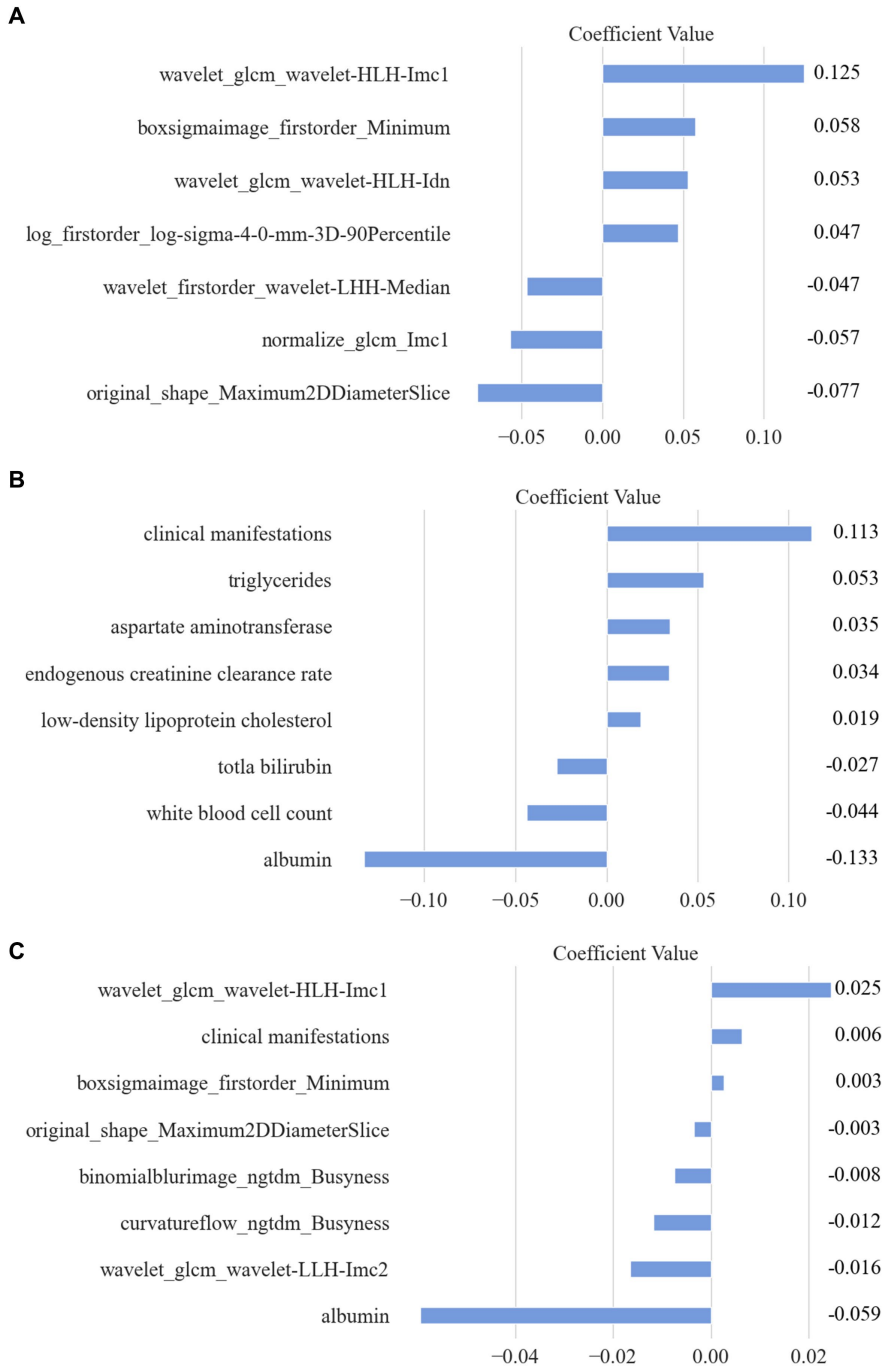
The selected features and their corresponding importance coefficients calculated by Lasso. **(A)** Selected Radiomic features. **(B)** Selected clinical features. **(C)** Combined features.

A total of 2,264 radiomic features were computed using the uAI Research Portal for each pancreas region. These features were then normalized using the *Z*-score approach. The feature selection method, Lasso, was employed to reduce dimensionality. Ultimately, 7-dimensional features were selected for the subsequent classification modeling. The names and corresponding importance coefficients of these 7 features calculated by Lasso are illustrated in Figure 4A. The selection of coefficient values was computed using the least square method. Each coefficient signifies the average impact of the corresponding feature on the selection results. In simpler terms, a higher value indicates a greater importance of the feature for the detection model.

Additionally, for each patient, a total of 24 clinical features were digitized and normalized using the *Z*-score method. Employing a similar feature selection procedure with Lasso, 8 clinical features were chosen. The names and coefficients of each clinical feature are featured in Figure 4B.

Moreover, a combination of all extracted radiomic features (2,264) and clinical features (24) resulted in a total of 2,288 features. Through Lasso computation, an optimal subset of 8 features was obtained, consisting of 6 radiomic features and 2 clinical features. Detailed information regarding all selected features is presented in Figure 4C.

### 3.2 Evaluation of models

The feature selection step automatically chose the most significant features for this classification task. In this step, all features were given equal priority and underwent *Z*-score normalization as a preprocessing step before using LASSO. To avoid overfitting, the LASSO parameter α was set to 0.05. The logistic regression classifier was then used to construct the detection model with the following parameter settings: a penalty factor C of 1.0, no class weight, L2 penalty type, a threshold of 0.5, and a tolerance of 1 × 10^−4^.

To effectively assess the performance of the detection models, the five-fold cross-validation technique was implemented due to the limited amount of data. Cross-validation is a widely accepted statistical technique used to evaluate predictive models by partitioning the original dataset into a training set to train the model, and a testing set to evaluate it. In the context of our study, we divided the complete dataset into five equal (or nearly equal) parts randomly. During the validation process, four of these subsets are combined to form the training set, and the remaining one subset is used as the testing set. This process is repeated five times (folds), with each of the five subsets serving as the testing set exactly once.

**Figure 5:**
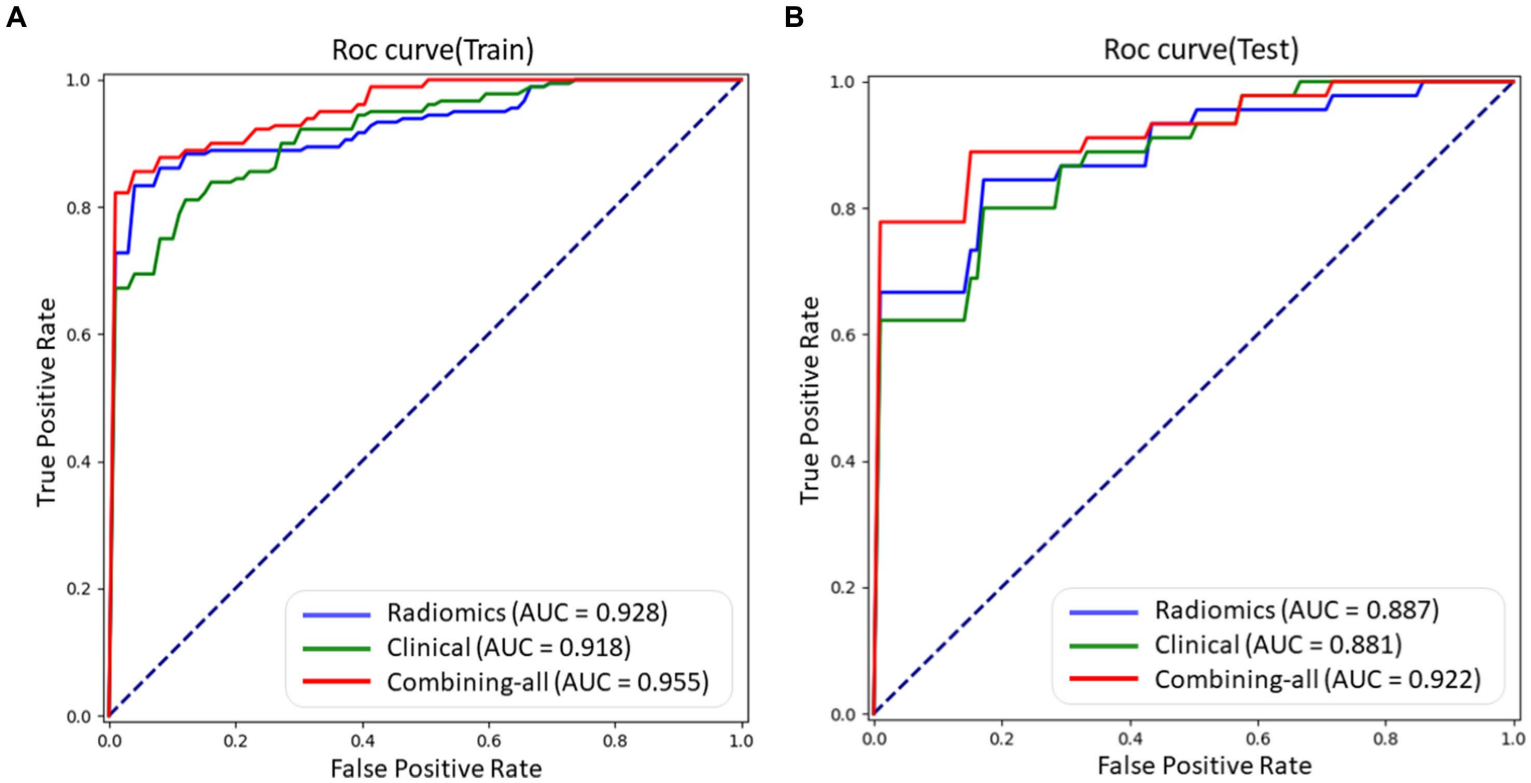
The ROC curves of different models using LR method, including radiomic features, clinical features, and combined features on train and test datasets, respectively. **(A)** ROC curve on train dataset. **(B)** ROC curve on test dataset.

LR, RF, and SVM classifiers were constructed for each fold using the selected features, which consisted of radiomic features, clinical features, and combined features. The overall performance was summarized by calculating the mean AUC, sensitivity, specificity, accuracy, and F1 score for both the training set and testing set. The results are presented in Table 2, we can found that LR get the best performance among these three models (RF, SVM, and Logistic) in AUC 0.922, Specificity 0.886, Accuracy 0.862, and F1 Score 0.871 indexes on test dataset with combined model, which demonstrate LR model having a robust classification ability. Then we do some other tests in the LR model, according to the Delong test, there was no statistically significant difference in the area under the ROC curve between the training set and testing set (as shown in Figure 5).

**Table 2:** The performance of the models built with radiomic features, clinical features, and combined features.

Methods	Methods	AUC (95% CI)	Sensitivity	Specificity	Accuracy	F1 score
**A. Performance of the models on train dataset**						
Clinical model	LR	0.918 (0.857–0.985)	0.85	0.795	0.827	0.85
	RF	0.871 (0.788–0.958)	0.778	0.788	0.782	0.805
	SVM	**0.96 (0.925–1)**	**0.933**	**0.812**	**0.882**	**0.901**
Radiomics model	LR	0.928 (0.87–0.993)	0.883	**0.894**	**0.888**	**0.901**
	RF	**0.945 (0.897–0.997)**	**0.894**	0.818	0.862	0.883
	SVM	0.861 (0.764–0.958)	0.833	0.749	0.798	0.827
Combined model	LR	0.955 (0.918–0.999)	0.861	**0.94**	**0.894**	0.904
	RF	**0.97 (0.94–1)**	**0.922**	0.879	0.904	**0.917**
	SVM	0.891 (0.801–0.981)	0.878	0.849	0.866	0.883
**B. Performance of the models on test dataset**						
Clinical model	LR	**0.881 (0.715–1)**	**0.822**	**0.757**	**0.795**	**0.819**
	RF	0.719 (0.441–0.967)	0.711	0.61	0.67	0.709
	SVM	0.876 (0.704–1)	0.8	0.752	0.782	0.807
Radiomics model	LR	**0.887 (0.73–1)**	**0.844**	**0.876**	**0.86**	**0.873**
	RF	0.794 (0.596–0.975)	0.822	0.638	0.745	0.779
	SVM	0.856 (0.679–1)	0.844	0.762	0.809	0.835
Combined model	LR	**0.922 (0.804–1)**	0.844	**0.886**	**0.862**	**0.871**
	RF	0.889 (0.748–1)	0.822	0.733	0.784	0.81
	SVM	0.902 (0.773–1)	**0.867**	0.767	0.823	0.85

The bold meaning is the best performance.

### 3.3 Regenerate response

The performance of the models built with radiomic features, clinical features, and combined features shown in Table 2.

## 4 Discussion

In our previous study (10), we calculated the fat fraction and CT value of the head, body, and tail of the pancreas from dual-energy CT images of 45 patients with type 2 diabetes mellitus (T2DM) and 33 control subjects. The experimental results demonstrated a significant association between the fat content of the pancreas and diabetes (10). While our focus was on fat fraction measurements, there are numerous other quantitative parameters that can be derived from dual-energy CT data. Hence, further studies could evaluate additional parameters such as radiomic features.

This retrospective study aims to establish a quantitative imaging biomarker for type 2 diabetes mellitus using dual-energy CT images of the pancreas. We have constructed three models using radiomic features, clinical features, and combined features, obtained through the LASSO regression approach, as shown in Table 3. The coefficients listed in Table 3 were derived from LASSO regression and represent the average impact of each corresponding feature on the classification results. A higher coefficient value indicates greater importance of the feature for the detection model.

**Table 3:** The significance of radiomic features, clinical features, and combined features to build three models.

Models	Features	Coefficient
Radiomics model	**wavelet_glcm_wavelet-HLH-Imc1**	**0.125**
	boxsigmaimage_firstorder_Minimum	0.057
	wavelet_glcm_wavelet-HLH-Idn	0.052
	log_firstorder_log-sigma-4-0-mm-3D-90Percentile	0.046
	wavelet_firstorder_wavelet-LHH-Median	−0.046
	**normalize_glcm_Imc1**	**−0.056**
	original_shape_Maximum2DDiameterSlice	−0.077
Clinical model	Clinical manifestations	0.112
	Triglycerides	0.053
	Aspartate aminotransferase	0.034
	Endogenous creatinine clearance	0.034
	LDL cholesterol	0.018
	Total bilirubin	−0.027
	White/ball	−0.043
	*albumin	−0.132
Combined model	**wavelet_glcm_wavelet-HLH-Imc1**	0.024
	Clinical manifestations	0.006
	boxsigmaimage_firstorder_Minimum	0.002
	original_shape_Maximum2DDiameterSlice	−0.003
	binomialblurimage_ngtdm_Busyness	−0.007
	curvatureflow_ngtdm_Busyness	−0.011
	**wavelet_glcm_wavelet-LLH-Imc2**	−0.016
	*albumin (clinical feature)	−0.059

The specific calculation method of each feature can be found from Pyradiomics website (https://pyradiomics.readthedocs.io/en/latest/features.html).

The bold meaning is the best performance.

The description and explanation of each feature for the three models are as follows: these radiomics features are linked to the focus of the doctor’s attention, such as image texture, gray level intensity, local homogeneity, and etc.

1. Informational Measure of Correlation (IMC) 1: IMC1 evaluates the correlation between the probability distributions of variables iandj , using mutual information $I\left(i,j\right)$ . This feature quantifies the complexity of the texture within the region of interest (ROI).
2. Minimum: This is a first-order feature that represents the minimum gray level intensity within the ROI.
3. Inverse Difference Normalized (IDN): IDN is a measure of the local homogeneity within the ROI.
4. 90 Percentile: This is a first-order feature that represents the number of voxels exceeding 90% of the voxel values in the set of all voxel values within the ROI.
5. Median: This first-order feature represents the median gray level intensity within the ROI.
6. Maximum 2D Diameter Slice: This shape feature is defined as the largest pairwise Euclidean distance between organ surface mesh vertices in the row-column plane (typically axial plane).

For the clinical model, diabetes is primarily characterized by hyperglycemia and metabolic disturbances. Its key clinical manifestations include increased appetite, excessive thirst, frequent urination, and unintended weight loss (i.e., more than three kilograms but less than one kilogram). In terms of serum biochemical markers, triglycerides, aspartate aminotransferase, endogenous creatinine clearance, low-density lipoprotein cholesterol, total bilirubin, and albumin are all intricately linked to human metabolism.

For the combined model, two additional features were listed as follows:

1. Informational Measure of Correlation (IMC) 2: IMC2 also evaluates the correlation between the probability distributions of variables iandj , similarly quantifying the complexity of the texture within the region of interest (ROI).
2. Busyness: This feature measures the change from a voxel to its neighbor. A high value for busyness indicates a “busy” image, with rapid changes in intensity between voxels and their neighborhood.

These additional features contribute to capturing more detailed information about texture complexity and local intensity variations in relation to diabetes detection.

From the selected features above, it is evident that some of them quantify the complexity of the texture of the pancreas. This suggests that there are rapid changes in intensity between voxels and their neighborhood in the medical images of patients with type 2 diabetes mellitus. These texture complexities may be indicative of underlying structural or compositional changes in the pancreas associated with diabetes. The identification and quantification of such changes can potentially provide valuable insights into disease progression and aid in the development of imaging biomarkers for diabetes diagnosis and monitoring.

Radiomics has indeed gained widespread utilization in clinical diagnosis. It has emerged as a valuable tool for auxiliary diagnosis by converting clinical images into mining data with high fidelity, repeatability, and minimal redundancy. This is achieved through the extraction of mathematical structural features from quantitative images. With the focus on personalized precision diagnosis and treatment, radiomics has played a pivotal role in advancing medical imaging beyond being just a diagnostic tool. It has become a fundamental instrument that provides specific and effective guidance for clinical diagnosis and treatment. By leveraging radiomic features, medical professionals can gain deeper insights into diseases, enabling them to make more accurate and personalized decisions regarding patient care (15). The integration of radiomics into clinical practice holds great promise for improving patient outcomes, optimizing treatment strategies, and facilitating precision medicine approaches.

Radiomics has been widely utilized in various diseases. van Griethuysen et al. (16) extracted radiomic features from 429 different lung lesions, including 48 texture features, 310 logarithmic features, and 158 wavelet features, to differentiate between benign and malignant nodules in the lungs. Their analysis revealed a correlation between image-based subtypes and the benign or malignant status of lung lesions. In another study, Grimm et al. (17) analyzed 275 preoperative breast MRI images of breast cancer patients and extracted a total of 56 imaging features encompassing morphology, texture, and dynamic characteristics for each patient’s image. Utilizing multivariate analysis, they determined the correlation between these imaging features and molecular subtypes of breast cancer. The findings revealed a significant association between radiomic features derived from dynamic contrast-enhanced MRI and the molecular subtypes of luminal A and luminal B hormone receptor-positive breast cancers.

Kaissis et al. (18) retrospectively analyzed preoperative CT images of 207 patients diagnosed with pancreatic ductal adenocarcinoma. They developed a random forest machine learning algorithm to accurately predict the molecular subtype of pancreatic cancer based on radiomic features. The classification algorithm demonstrated high sensitivity (0.84 ± 0.05) and specificity (0.92 ± 0.01). Furthermore, the area under the receiver operating characteristic curve (AUC-ROC) was determined to be 0.93 ± 0.01, indicating that preoperative CT image radiomics analysis utilizing machine learning holds promise in predicting molecular subtypes closely associated with the survival outcomes of pancreatic cancer patients. Xue et al. (19) retrospectively analyzed CTA images and clinical data of 170 cases involving the head and radiomic features in conjunction with elevated levels of homocysteine and hypertension. Both the Rad-score model and joint model were established to investigate associations with symptomatic carotid plaque. Their findings demonstrated that hyper-homocysteinemia and hypertension exhibited independent associations with symptomatic carotid plaque. These studies highlight the potential of radiomics in various disease contexts, including lung lesions, breast cancer, pancreatic cancer, and carotid plaque. Radiomics analysis can provide valuable insights into disease characterization, subtype classification, and prediction of clinical outcomes.

In this study, a total of 2,264 radiomic features were extracted from pancreatic dual-energy CT images. A feature selection procedure similar to Lasso was employed to identify 7 optimal features. Simultaneously, 24 clinical features were digitized and normalized. Out of these, 8 representative clinical features were selected. The radiomic features were integrated with the clinical features, resulting in an optimal subset comprising of 8 calculated features determined by the Lasso algorithm. Subsequently, three models were constructed: one solely based on radiomic features, another solely based on clinical features, and the third model incorporating both types of features. The performance evaluation of these models was conducted using cross-validation. In the test set, the area under the curve (AUC) values were 0.887 (0.73–1), 0.881 (0.715–1), and 0.922 (0.804–1), respectively. When compared with previous studies (10), the sensitivity (0.844, 0.822, 0.844), specificity (0.876, 0.757, 0.886), accuracy (0.86, 0.795, 0.862), and F1 score (0.873, 0.819, 0.871) of this study showed significant improvement in performance.

**Figure 6:**
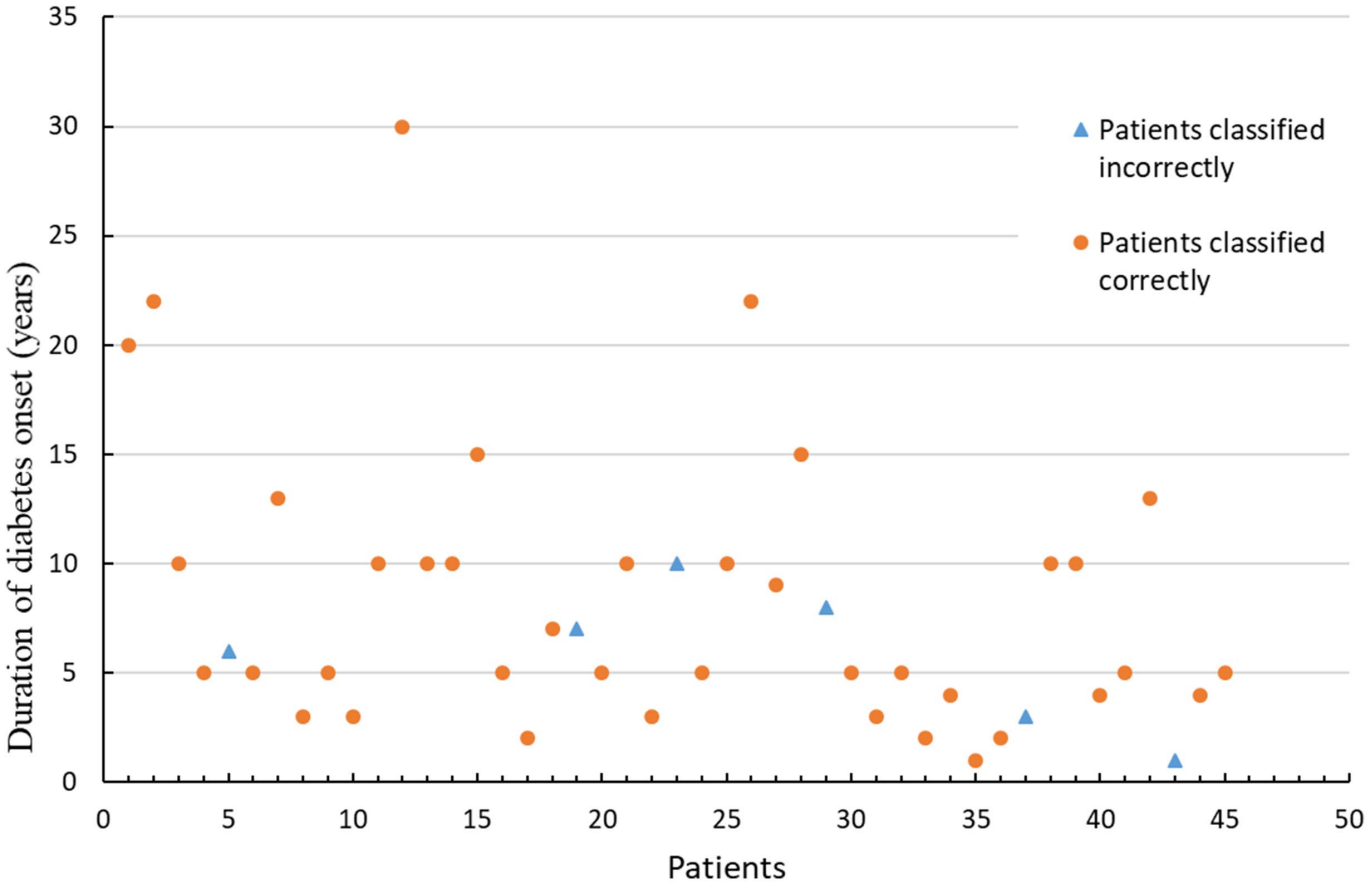
Statistical graph of the disease duration for 45 diabetes patients.

Furthermore, we evaluated the performance of the combined model using three clinical features: average blood glucose, duration of diabetes, and diabetic complications. The feature “average blood glucose” was used for the diagnosis of diabetes patients, which is consistent with the ground truth. Regarding diabetes duration, all patients who were classified incorrectly by the combined model had a disease duration of less than 10 years. This interesting finding may be due to the selected feature not being able to capture the difference between healthy controls and diabetes patients with a duration of less than 10 years as shown in Figure 6. Additionally, when classifying patients with (25 patients) or without (20 patients) diabetic complications, 2 patients were classified into the complication group.

There are several limitations to our study. Firstly, the sample size was relatively small, and further expansion of the sample size is necessary to enhance the credibility of the results. Secondly, we only analyzed the imaging features of all patients using dual-energy pancreatic plain scans without considering differences in pancreatic imaging features during different enhanced phases. Additionally, we solely selected axial pancreatic images for analysis, potentially resulting in the omission of certain characteristic radiomics data.

## 5 Conclusion

After a comprehensive analysis, the three models constructed based on CT radiomic features demonstrate the potential of pancreatic dual-energy CT images as quantitative imaging biomarkers for detecting type 2 diabetes.

## Data availability statement

The raw data supporting the conclusions of this article will be made available by the authors, without undue reservation.

## Ethics statement

Ethical approval was not required for the study involving humans in accordance with the local legislation and institutional requirements. Written informed consent to participate in this study was not required from the participants or the participants’ legal guardians/next of kin in accordance with the national legislation and the institutional requirements.

## Author contributions

WJ: Writing – original draft, Data curation, Formal analysis, Investigation, Methodology, Project administration, Resources, Writing – review & editing. XP: Writing – original draft, Data curation, Formal analysis, Methodology, Software, Writing – review & editing. QL: Writing – original draft, Writing – review & editing. SH: Writing – original draft, Writing – review & editing. YuL: Writing – original draft, Writing – review & editing. XiZ: Writing – original draft, Writing – review & editing. XeZ: Writing – original draft, Writing – review & editing. WD: Writing – original draft, Writing – review & editing. YaL: Writing – original draft, Writing – review & editing. LC: Writing – original draft, Writing – review & editing.

## References

[1] Marshall SM. The pancreas in health and in diabetes. Diabetologia. (2020) 63:1962–5. doi: 10.1007/s00125-020-05235-z 32894305

[2] Garcia TS Rech TH Leitão CB. Pancreatic size and fat content in diabetes: a systematic review and meta-analysis of imaging studies. PLoS One. (2017) 12:e0180911. doi: 10.1371/journal.pone.0180911, PMID: 28742102 PMC5524390

[3] Yamazaki H Tauchi S Wang J Dohke M Hanawa N Kodama Y. Longitudinal association of fatty pancreas with the incidence of type-2 diabetes in lean individuals: a 6-year computed tomography-based cohort study. J Gastroenterol. (2020) 55:712–21. doi: 10.1007/s00535-020-01683-x, PMID: 32246380

[4] Tallam H Elton DC Lee S Wakim P Pickhardt PJ Summers RM. Fully automated abdominal CT biomarkers for type 2 diabetes using deep learning. Radiology. (2022) 304:85–95. doi: 10.1148/radiol.211914, PMID: 35380492 PMC9270681

[5] Sofue K Itoh T Takahashi S Schmidt B Shimada R Negi N. Quantification of cisplatin using a modified 3-material decomposition algorithm at third-generation dual-source dual-energy computed tomography: an experimental study. Investig Radiol. (2018) 53:673–80. doi: 10.1097/RLI.0000000000000491, PMID: 29912043

[6] Zhou Y Zhang J Chen J Yang C Gong C Li C. Prediction using T2-weighted magnetic resonance imaging-based radiomics of residual uterine myoma regrowth after high-intensity focused ultrasound ablation. Ultrasound Obstet Gynecol. (2022) 60:681–92. doi: 10.1002/uog.26053 36054291 PMC9828488

[7] An C Kim DW Park YN Chung YE Rhee H Kim MJ. Single hepatocellular carcinoma: preoperative MR imaging to predict early recurrence after curative resection. Radiology. (2015) 276:433–43. doi: 10.1148/radiol.15142394 25751229

[8] Martin SS Trapp F Wichmann JL Albrecht MH Lenga L Durden J. Dual-energy CT in early acute pancreatitis: improved detection using iodine quantification. Eur Radiol. (2019) 29:2226–32. doi: 10.1007/s00330-018-5844-x, PMID: 30488112

[9] Wright DE Mukherjee S Patra A Khasawneh H Korfiatis P Suman G. Radiomics-based machine learning (ML) classifier for detection of type 2 diabetes on standard-of-care abdomen CTs: a proof-of-concept study. Abdom Radiol. (2022) 47:3806–16. doi: 10.1007/s00261-022-03668-1, PMID: 36085379 PMC12315806

[10] Huang S Liang Y Zhong X Luo Q Yao X Nong Z. Pancreatic fat fraction in dual-energy computed tomography as a potential quantitative parameter in the detection of type 2 diabetes mellitus. Eur J Radiol. (2023) 159:110668. doi: 10.1016/j.ejrad.2022.110668, PMID: 36608599

[11] Shi F Hu W Wu J Han M Wang J Zhang W. Deep learning empowered volume delineation of whole-body organs-at-risk for accelerated radiotherapy. Nat Commun. (2022) 13:6566. doi: 10.1038/s41467-022-34257-x, PMID: 36323677 PMC9630370

[12] Wu J Xia Y Wang X Wei Y Liu A Innanje A. uRP: an integrated research platform for one-stop analysis of medical images. Front Radiol. (2023) 3:1153784. doi: 10.3389/fradi.2023.1153784, PMID: 37492386 PMC10365282

[13] Zheng BH Liu LZ Zhang ZZ Shi JY Dong LQ Tian LY. Radiomics score: a potential prognostic imaging feature for postoperative survival of solitary HCC patients. BMC Cancer. (2018) 18:1–12. doi: 10.1186/s12885-018-5024-z, PMID: 30463529 PMC6249916

[14] Harrell FE Jr Lee KL Califf RM Pryor DB Rosati RA. Regression modelling strategies for improved prognostic prediction. Stat Med. (1984) 3:143–52. doi: 10.1002/sim.4780030207 6463451

[15] Feng XY. Precision medicine, medical imaging first. Natl Med J China. (2016) 1:1–2. doi: 10.3760/cma.j.issn.1005-1201.2016.01.001

[16] van Griethuysen JJM Fedorov A Parmar C Hosny A Aucoin N Narayan V. Computationalradiomics system to decode the radiographic pheno type. Cancer Res. (2017) 77:e104–7. doi: 10.1158/0008-5472.CAN-17-0339, PMID: 29092951 PMC5672828

[17] Grimm LJ Zhang J Mazurowski MA. Computational approach to radiogenomics of breast cancer: luminal A and luminal B molecular subtypes are associated with imaging features on routine breast MRI extracted using computer vision algorithms. J Magn Reson Imaging. (2015) 42:902–7. doi: 10.1002/jmri.24879, PMID: 25777181

[18] Kaissis GA Ziegelmayer S Lohofer FK Harder FN Jungmann F Sasse D. Image-based molecular phenotyping of pancreatic ductal adenocarcinoma. J Clin Med. (2020) 9:724. doi: 10.3390/jcm9030724, PMID: 32155990 PMC7141256

[19] Xue LL Wang LJ Shi CY Zhang Q Qiao Y Zhang H. Radiomics analysis of CTA for identification of symptomatic carotid artery plaques. Chin J Integr Med Cardio/Cerebrovasc Dis. (2023) 21:2083–8. doi: 10.12102/j.issn.1672-1349.2023.11.032

