# Supplementary material for: Radiomics analysis of pancreas based on dual-energy computed tomography for the detection of type 2 diabetes mellitus
Source: Front Med (Lausanne). 2024 Apr 19;11:1328687. doi: 10.3389/fmed.2024.1328687 (PMC11069320; doi:10.3389/fmed.2024.1328687)
Supplement: Supplementary file 1 [file Data_Sheet_1.PDF]

Supplementary Tables

Table S1. The extracted texture features in this study.

Gray Level Co-occurrence Matrix (GLCM, n=24)	The GLCM functions characterize the texture of an image by calculating how often pairs of pixel with specific values and in a specified spatial relationship occur in an image, creating a GLCM, and then extracting statistical measures from this matrix.
Gray Level Run Length Matrix Features (GLRLM, n=16)	GLRLM quantifies gray level runs, which are defined as the length in number of pixels, of consecutive pixels that have the same gray level value.
Gray Level Size Zone Matrix (GLSZM, n=16)	GLSZM quantifies gray level zones in an image. A gray level zone is defined as a number of connected voxels that share the same gray level intensity. A voxel is considered connected if the distance is 1 according to the infinity norm (26-connected region in a 3D, 8-connected region in 2D).
Neighboring Gray Tone Difference Matrix (NGTDM, n=5)	A Neighboring Gray Tone Difference Matrix quantifies the difference between a gray value and the average gray value of its neighbors within distance δ. The sum of absolute differences for gray level i is stored in the matrix.
Gray Level Dependence Matrix (GLDM, n=14)	A Gray Level Dependence Matrix (GLDM) quantifies gray level dependencies in an image. A gray level dependency is defined as a the number of connected voxels within distance δ that are dependent on the center voxel.

Table S2. The selected features 'Imc1', 'minimum', and 'Idn' in Table 3 can be described as follows.

Feature	Definition	Definition
wavelet_glcm_wavelet-HLH-Imc1	The term "wavelet" indicates the type of filter used, "glcm" represents the feature group being analyzed, and the final term refers to the specific feature being extracted.	Informational Measure of Correlation (IMC) 1: IMC1 evaluates the correlation between the probability distributions of variables i and j, using mutual information I(i,j). This feature quantifies the complexity of the texture within the region of interest (ROI).
boxsigmaimage_firstorder_Minimum	The specific filter used is 'box sigma image', the feature group being analyzed is 'first order', and the computed feature is 'minimum'.	This is a first-order feature that represents the minimum gray level intensity within the ROI.
wavelet_glcm_wavelet-HLH-Idn	The used filter is 'wavelet', the feature group is 'glcm', and the computed feature is 'idn'. These calculations are based on the results filtered by 'wavelet-HLH'.	Inverse Difference Normalized (IDN): IDN is a measure of the local homogeneity within the ROI.
